# Mapping Clinical Intent in Internet Hospitals: Identifying and Profiling Patient Demand via Natural Language Processing

**DOI:** 10.34133/csbj.0132

**Published:** 2026-06-05

**Authors:** Ruoxin Xu, Jing Liu, Huizi Ye, Tingting Yang, Kuanlie Jiang, Xiaojiao Zhu

**Affiliations:** ^1^ Yuebei People’s Hospital Affiliated to Shantou University Medical College, Shaoguan, Guangdong, China.; ^2^ Hainan Medical University, Haikou, Hainan, China.

## Abstract

Patient-generated text in internet hospitals represents a massive but unstructured source of clinical demand. Misalignment between rigid administrative menus and actual patient intent leads to triage inefficiency and resource mismatches. We developed a hybrid artificial intelligence framework to decode patient needs and optimize service allocation using real-world data from a tertiary internet hospital, with external validation on an independent multihospital dataset (*N* = 497). In this study, we analyzed 28,851 inquiries from a tertiary internet hospital using a 2-stage approach: (a) unsupervised K-Means clustering to discover intrinsic intent taxonomies, and (b) supervised classification using a fine-tuned MacBERT model. We benchmarked this specialized small model against traditional baselines and state-of-the-art large language models (LLMs; Qwen2.5 and HuatuoGPT-II) on a gold-standard dataset of 4,814 annotated records. Unsupervised analysis identified 8 distinct patient intents, revealing substantial semantic overlap between rehabilitation and follow-up requests. The fine-tuned MacBERT model achieved a weighted F1-score of 0.680, outperforming both the medical-specific LLM (F1 = 0.595) and the general LLM (F1 = 0.585). External validation on an independent nationwide platform dataset (*N* = 497) confirmed cross-platform transferability, with the hybrid learning framework achieving a weighted F1 of 0.578 versus 0.389 (Qwen2.5-7B) and 0.190 (HuatuoGPT-II). Departmental analysis exposed distinct “demand fingerprints”: 39% of Neurology inquiries focused on prescription renewals, whereas 68% of Dermatology inquiries involved initial symptom consultations. These findings indicate that, within the studied Chinese hospital setting, fine-tuned task-specific models may offer superior utility over general LLMs for clinical triage, with the performance gap widening under domain shift—though multicenter prospective validation is required to establish generalizability. Mapping intent fingerprints across specialties provides a quantifiable basis for restructuring digital hospital operations.

## Graphical Abstract

**Figure:**
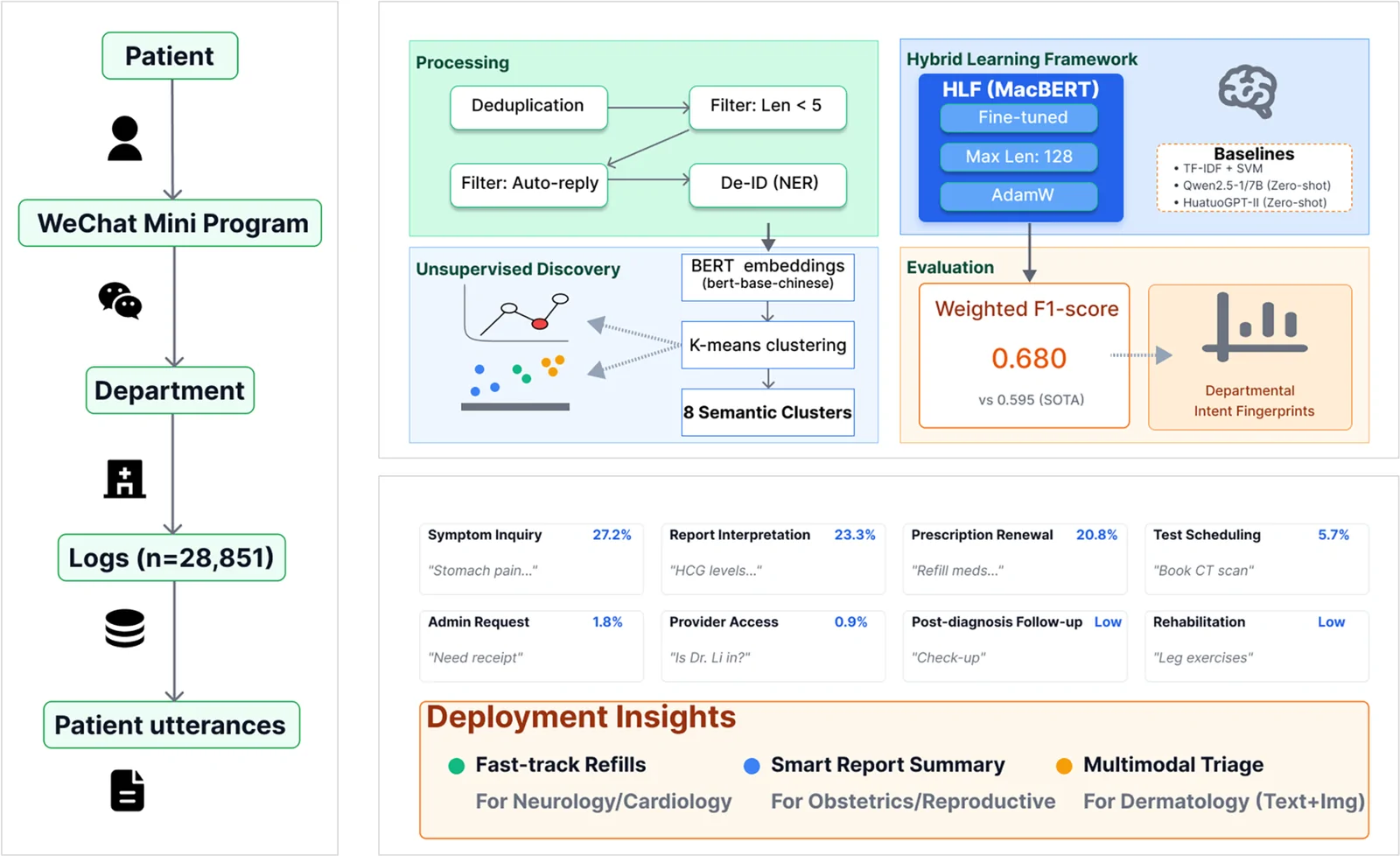

## Introduction

Inefficient patient access orchestration ranks among the primary operational bottlenecks in digital health ecosystems globally, and its impact is particularly acute in internet hospitals, where elevated triage failure rates pose a considerable burden on regional healthcare systems [1], just as diagnostic delays directly compromise patient survival and disease progression [2] and inappropriate referrals to specialized services serve as a primary driver for systemic inefficiency and prolonged waiting times [3]. The structural misalignment between patient narratives and hospital taxonomies is an important modifiable risk factor for healthcare operational inefficiency. The World Health Organization has identified digital health optimization as a cost-effective strategy for averting resource waste and improving service accessibility [4]. However, well into the digital era, the operational reality is that patient self-triage failure rates remain high. Systematic reviews indicate that primary diagnostic accuracy can be as low as 19%, while triage accuracy fluctuates unpredictably between 49% and 90% depending on the condition, leading to mismatch rates that frequently exceed acceptable thresholds for patient safety [5]. For instance, retrospective audits in tertiary centers reveal that up to 15% of patients registering for cardiology services are actually seeking routine prescription refills better suited for general practice. Dermatology channels, similarly, are frequently occupied by nonurgent inquiries that delay acute care responses. Patients navigating these platforms often face challenges such as limited medical literacy and reduced access to guidance, leading to frequent departmental mismatches that compromise care delivery [6].

Lack of systemic capability to decode the major sources of intent in patient narratives perpetuates this operational inefficiency. Of particular concern is the systemic blindness to hybrid intents (complex inquiries where patients weave clinical symptoms with logistical or financial constraints) and to long-tail query patterns. As highlighted in recent dialogue synthesis research, such data imbalance and scarcity of low-resource training samples severely hinder model accuracy for diverse patient needs [7]. In typical internet hospital logs, the vast majority of inquiries come from colloquial, unstructured expression—a format that is notoriously difficult to process automatically and remains largely untapped compared to structured medical terminology [8]. A patient might simultaneously describe a stomach ache (clinical symptom), ask about “insurance coverage” (administrative rule), and request “an appointment for tomorrow” (logistical constraint). Furthermore, while this is a long-standing problem in general healthcare, it is increasingly true in high-throughput digital platforms where the volume of inquiries exceeds human processing capacity [1]. This lack of interpretative capability is aggravated by some prospective studies reporting precision rates as low as 33% for critical triage levels [9]. Most traditional natural language processing (NLP) initiatives are often de-prioritized or underperform because they rely on datasets that fail to reflect the reality of patient-initiated inquiries—often producing generic responses that lack specific clinical reasoning [10]. Methodological research has predominantly relied on 2 polarized data sources: social media text (e.g., Reddit and Twitter), which is primarily utilized for sentiment surveillance rather than actionable clinical triage [11], or Medical Information Mart for Intensive Care III (MIMIC-III) clinical notes, which represent professional medical synthesis rather than raw patient expression [12]. This global drop-off in domain-specific model performance occurred in parallel with a global rise in strategies focusing on generic large models, leaving a vacuum in tools designed specifically for the “digital front door”, defined here as the initial patient-facing interface where triage and routing decisions first occur. Recent advancements like ChatDoctor demonstrate that domain-specific tuning is necessary to meet the low error tolerance required in such medical interactions [13].

Historically, it has also been challenging to effectively automate triage and measure its impact in workflows with high noise and semantic ambiguity [14]. While population-based artificial intelligence (AI) initiatives have shown promise, disseminating these equitably, to meet the specific operational logic of individual hospitals, has remained challenging [15]. The recent rise of online health information-seeking and large language models (LLMs) opens new pathways [16], but even for some advanced models, marked hallucination disparities have been observed [17]. Interestingly, the operational requirements of a public hospital differ from the general reasoning capabilities driven by LLM benchmarks. Generalist models are more likely to invent nonexistent protocols, such as booking channels that do not exist, or cite refund policies that contradict actual regulations than to strictly adhere to local rules [18]. Moreover, the deployment of cloud-based models introduces important data security vulnerabilities and privacy concerns, which severely compromise patient trust and are operationally unacceptable for sensitive routing systems [19]. This body of evidence culminates in calls to researchers to produce scalable, domain-specific models so that even resource-constrained hospitals can benefit from them [20]. There is an urgent, unmet need for architectures that decouple reasoning capability from model size. Domain-specialized agents, such as those optimized for biomedical contexts [21] or enhanced with whole-word masking strategies [22], may offer a superior cost–benefit ratio for specific triage tasks, but this hypothesis remains to be empirically tested. The COVID-19 pandemic catalyzed the emergence of an innovative approach to health communication that was both easy to understand and rapidly scalable: the internet hospital [23,24]. In this study, we systematically evaluate the effect of a hybrid learning framework (HLF) designed to convey essential understanding of patient intent on triage accuracy and operational efficiency (Fig. 1).

**Fig. 1.**
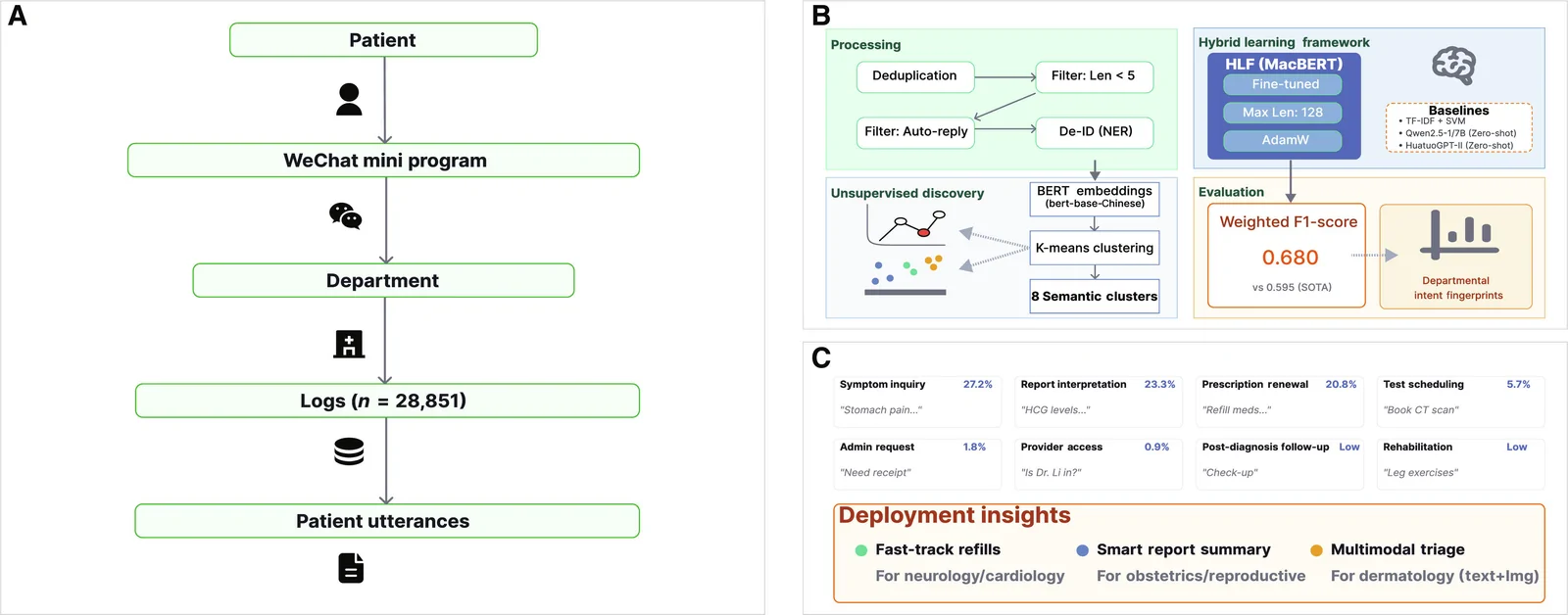
Overview of the study design, technical framework, and clinical applications. (A) Data acquisition pathway: Patient-generated text data (*N* = 28,851) were collected via the WeChat Mini Program from a tertiary hospital, capturing the complete digital journey from login to consultation. (B) Hybrid learning framework (HLF): The technical pipeline consists of 3 phases: (a) Preprocessing to ensure data hygiene and privacy (de-identification); (b) unsupervised discovery using BERT embeddings and K-Means clustering (validated by the Elbow Method and t-SNE) to uncover the intrinsic taxonomy; and (c) supervised learning, where a fine-tuned MacBERT model (HLF) is trained on a gold-standard dataset (*N* = 4,814) and benchmarked against baselines (SVM, Qwen2.5, and HuatuoGPT-II). (C) Taxonomy and operational insights: The model identified 8 distinct patient intents (e.g., Initial Symptom Inquiry, 27.2%). The right panel highlights 3 data-driven operational strategies derived from these patterns: fast-tracking refills for chronic care, AI-summarized reports for obstetrics, and multimodal triage for dermatology.

Beyond pandemic-related urgency, recent trials have demonstrated the potential for specialized AI to increase service precision and behavioral intent to adopt digital health recommendations [25–27]. This research will contribute to the existing literature on medical NLP and inform future scalable strategies aimed at delivering precise digital health services. Distinct from approaches that force data into preexisting administrative categories, our framework first integrates unsupervised clustering to reveal the latent taxonomy of patient demands. Subsequently, we engineer a lightweight classifier specifically designed to bridge the gap between colloquial narratives and institutional resources. Ultimately, these results could guide system-wide initiatives that use data-driven taxonomies to improve access efficiency and reduce resource mismatch.

This study aimed to:

1. Systematically mine the latent taxonomy of patient demands from unstructured logs using unsupervised clustering, addressing the lack of ground-truth labels.
2. Measure the effect of a fine-tuned small language model (MacBERT) on triage accuracy compared to generalist LLMs under zero-shot conditions, and assess whether domain-specific fine-tuning confers robustness to cross-platform distributional shifts.
3. Map the “demand fingerprints” of clinical departments to inform operational strategy and optimize resource allocation tailored to actual patient demand.

We hypothesized that the specialized HLF model tested here would increase triage accuracy significantly compared to generalist baselines, with the expectation that domain-specific fine-tuning is critical for bridging the semantic gap in patient narratives [28].

## Results

### Dataset characteristics and taxonomy validation

The gold-standard dataset for supervised learning comprised 4,814 patient–physician interaction records, randomly sampled from the full corpus (N=28,851). Analysis of intent distribution revealed a pronounced long-tail pattern characteristic of digital health platforms.

Initial Symptom Inquiry emerged as the predominant category, comprising 27.2% (n=1,360) of the total annotated dataset. This high frequency reflects the primary role of the internet hospital as a digital front door for patients seeking preliminary medical advice. The second and third most frequent categories were Test Report Interpretation (23.3%, n=1,165) and Prescription Renewal (20.8%, n=1,040). These 3 categories cumulatively account for over 71% of the total inquiry volume, indicating that the majority of patient needs are concentrated in consultation, result reading, and medication management.

In contrast, the dataset exhibited a long tail of administrative and logistical inquiries. Test Scheduling and Ordering (n=285, 5.7%) and Administrative Request (n=90, 1.8%) were relatively sparse. Specific Provider Access was the rarest category, representing only 0.9% (n=45) of the data. Inter-annotator agreement, assessed on a randomly selected subset, yielded a Cohen’s Kappa coefficient of 0.88, indicating strong consensus on the taxonomy definitions.

To validate the intrinsic dimensionality of the dataset structure, we applied the Elbow Method to the BERT embeddings. The plot of the sum of squared errors (SSE) against the number of clusters (*k*) exhibited a distinct inflection point at *k* = 8 (Fig. 2A). This suggests that increasing *k* beyond 8 yields diminishing returns in variance explanation, providing empirical support for the adoption of an 8-category schema.

**Fig. 2.**
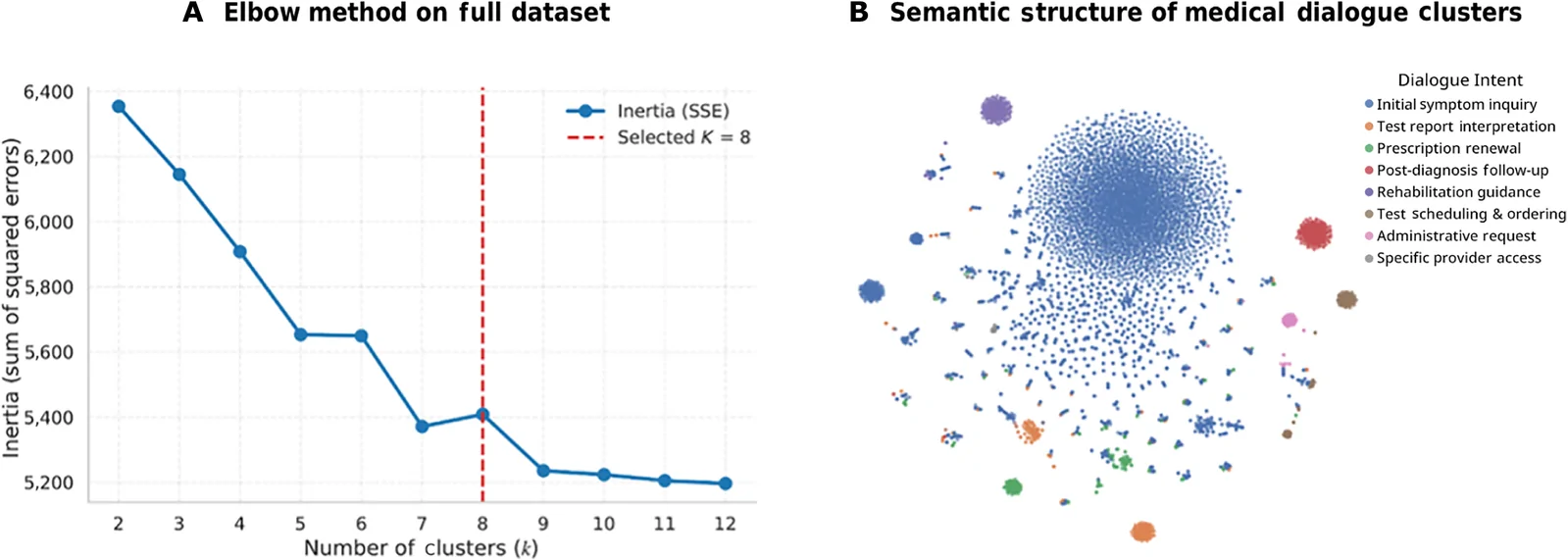
Unsupervised validation of the intent taxonomy. (A) Determination of the optimal number of clusters using the Elbow Method. The sum of squared errors (SSE) shows a distinct inflection point at *k* = 8 (red dot), indicating diminishing returns for additional clusters. (B) t-SNE visualization of the semantic space of patient inquiries. Each point represents a single patient query. The clear separation among the 8 color-coded clusters validates that the taxonomy is rooted in the natural semantic structure of the data.

To further corroborate this structure, we employed t-Distributed Stochastic Neighbor Embedding (t-SNE) to project the high-dimensional BERT embeddings into a 2-dimensional semantic space (Fig. 2B). The visualization revealed 8 distinct, dense clusters corresponding to the defined intents. For instance, inquiries related to Prescription Renewal formed a tight, isolated cluster, indicating high semantic consistency. However, the plot also revealed semantic proximity between the Post-diagnosis Follow-up and Rehabilitation Guidance clusters, foreshadowing the classification challenges observed in the supervised phase.

To rigorously define the semantic meaning of each cluster, we analyzed the top keywords extracted via Term Frequency–Inverse Document Frequency (TF-IDF). As illustrated in Fig. 3, distinct lexical patterns emerged for each group. For example, Cluster C3 is dominated by terms like Prescribe Meds, Diabetes, and Hypertension, clearly indicating a Prescription Renewal intent. Similarly, Cluster C0 is characterized by Report and CT Scan, defining the Test Report Interpretation category. Based on these keyword signatures, we formally defined the 8-category taxonomy used for subsequent annotation.

**Fig. 3.**
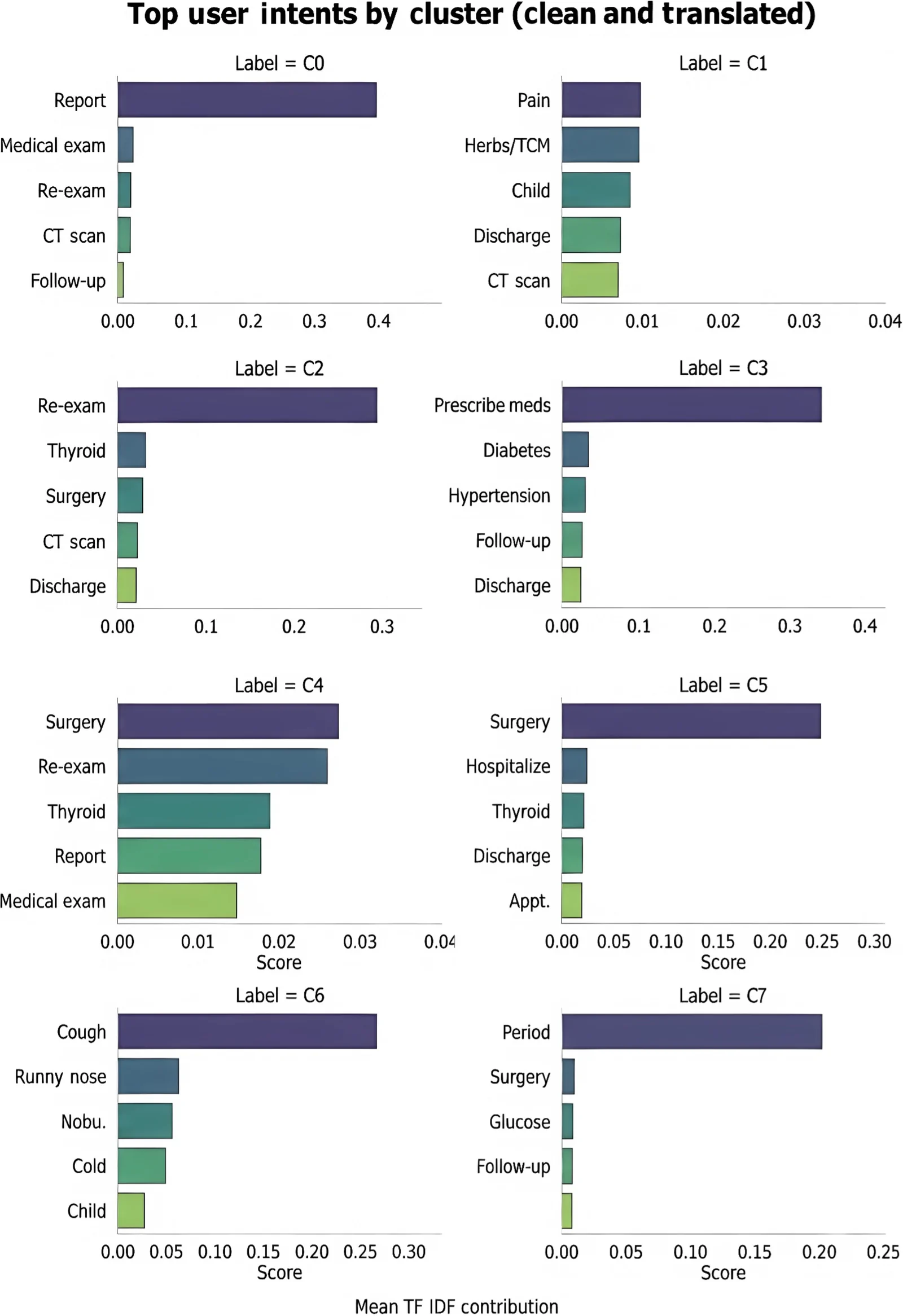
Top TF-IDF keywords for each of the 8 clusters (C0 to C7). Keywords serve as the interpretative basis for defining the clinical intent categories, as described in the text.

### Comparative benchmarking of model architectures

A comprehensive performance comparison of the proposed HLF against 3 tiers of baseline models (traditional machine learning, general LLMs, and medical LLMs) is presented in Table 1.

**Table 1.** Performance comparison of different models on patient intent classification. The proposed method (hybrid learning framework [HLF]) is benchmarked against traditional baselines (support vector machine [SVM]), general large language models (Qwen2.5-7B), and medical-specific large language models (LLMs; HuatuoGPT-II). All reported metrics are weighted averages. Bootstrap resampling (1,000 iterations) yielded a 95% confidence interval of [0.663, 0.692] for the HLF weighted F1. McNemar tests confirmed that the HLF significantly outperformed all baseline models on the in-domain test set (*P* < 0.001 for all pairwise comparisons). Bold values indicate the best performance. The LLM baselines were evaluated in a zero-shot setting; their performance under few-shot or fine-tuned configurations may differ.

Model category	Model architecture	Parameters	Setup	Accuracy	Precision	Recall	Macro F1	Weighted F1
Traditional ML	TF-IDF + SVM	–	Baseline	0.421	0.443	0.421	0.315	0.393
General LLMs	Qwen2.5-1.5B	1.5B	Zero-shot	0.49	0.534	0.49	0.412	0.473
	Qwen2.5-7B	7B	Zero-shot	0.602	0.598	0.602	0.528	0.585
Medical LLM	HuatuoGPT-II	7B	Zero-shot	0.62	0.602	0.62	0.541	0.595
**Proposed framework**	**HLF (MacBERT)**	**0.1B**	**Fine-tuned**	**0.692**	**0.71**	**0.67**	**0.615**	**0.68**

The traditional baseline (TF-IDF + SVM [support vector machine]) demonstrated the severe limitations of noncontextual methods in medical NLP. It achieved a weighted F1-score of only 0.393, with a particularly low recall of 0.421. The TF-IDF + SVM baseline achieved a weighted F1-score of 0.393 and a recall of 0.421, indicating limited capacity to handle the lexical diversity of patient-generated text.

In the realm of generative AI, the general LLM (Qwen2.5-7B), evaluated in a zero-shot setting, showed a marked improvement over the SVM baseline, achieving a weighted F1-score of 0.585. This indicates that large-scale pretraining endows the model with a baseline level of capability to understand medical intents.

Interestingly, the medical-specific LLM (HuatuoGPT-II) provided only a marginal performance gain over the general LLM. Despite being pretrained on massive Chinese biomedical corpora, it achieved a weighted F1-score of 0.595, outperforming the general Qwen model by only 1.0 percentage point. This finding implies that general medical knowledge alone does not guarantee high accuracy in specific hospital administrative tasks.

Most notably, our HLF, based on the fine-tuned MacBERT model, outperformed all baselines by a substantial margin. The HLF achieved a weighted F1-score of 0.680, surpassing the specialized medical LLM by 8.5 percentage points and the traditional baseline by nearly 29 percentage points. In terms of Precision, the HLF reached 0.710, compared to 0.602 for HuatuoGPT-II. The average inference time per inquiry was 6.2 ms on an NVIDIA RTX 3090 GPU, well within the latency requirements of real-time clinical triage.

### Cross-institutional external validation

To determine whether the HLF’s performance advantage persists beyond the training institution, we applied the trained model directly to the independent Haodf dataset (*N* = 497) without any retraining or adaptation. The Haodf platform differs from the primary study site in 3 key respects: (a) it serves patients nationwide through a web-based interface rather than a single-hospital WeChat Mini Program; (b) its user base skews toward first-contact consultation rather than chronic disease management; and (c) its inquiry style reflects a public portal rather than an institution-specific workflow. These differences jointly constitute a meaningful test of domain shift.

Table 2 summarizes the cross-institutional results. The HLF model achieved a weighted F1-score of 0.582 on Haodf, representing a decline of 9.8 percentage points relative to its in-domain performance (0.680). This domain gap is consistent with expectations for clinical NLP under platform-level distributional shifts. More notably, the domain shift disproportionately affected the zero-shot LLM baselines. Qwen2.5-7B dropped to a weighted F1 of 0.389 (−19.6 pp from in-domain), and the medical-specific HuatuoGPT-II fell to 0.390 (−20.5 pp), a level approaching random guessing for an 8-class task. Consequently, the HLF’s performance margin over the LLM baselines widened substantially under domain shift—from 8.5 percentage points in-domain (*vs.* HuatuoGPT-II) to 19.2 percentage points cross-domain.

**Table 2.** Cross-institutional performance of the hybrid learning framework on an independent external dataset. The HLF, trained exclusively on the primary hospital dataset, is evaluated on an independent nationwide platform dataset (Haodf, *N* = 497) alongside Qwen2.5-7B and HuatuoGPT-II under zero-shot conditions. Δ denotes absolute change in weighted F1 from in-domain to cross-domain evaluation. Negative values indicate performance decline. Bootstrap 95% confidence intervals (1,000 iterations) on Haodf: HLF [0.535, 0.630]; Qwen2.5-7B [0.346, 0.440]; HuatuoGPT-II [0.349, 0.431]. McNemar tests confirmed that the HLF significantly outperformed both LLM baselines (*P* < 0.001 for both comparisons). The HLF model weights are held fixed from the primary hospital training set; both LLMs are evaluated without fine-tuning or few-shot exemplars.

Model	Parameters	Setup	In-domain weighted F1	Cross-domain weighted F1	Δ (pp)
HLF (MacBERT)	0.1B	Fine-tuned	0.680	0.582	−9.8
Qwen2.5-7B	7B	Zero-shot	0.585	0.389	−19.6
HuatuoGPT-II	7B	Zero-shot	0.595	0.390	−20.5

These results indicate that the HLF’s learned decision boundaries, while optimized on single-center data, carry discriminative information that transfers across institutional and platform boundaries. In contrast, zero-shot LLM inference—despite the breadth of pretraining corpora—is brittle to distributional shifts in patient language, platform conventions, and inquiry formulation. This finding reinforces the practical argument for task-specific fine-tuning in operational clinical NLP deployments. Bootstrap 95% confidence intervals for the 3 models were nonoverlapping on the external dataset, and McNemar tests confirmed that the HLF significantly outperformed both LLM baselines (*P* < 0.001 for both comparisons).

### Category-specific performance and error analysis

To provide a granular understanding of the model’s capabilities, we analyzed performance at the individual category level using the confusion matrix (Fig. 4).

**Fig. 4.**
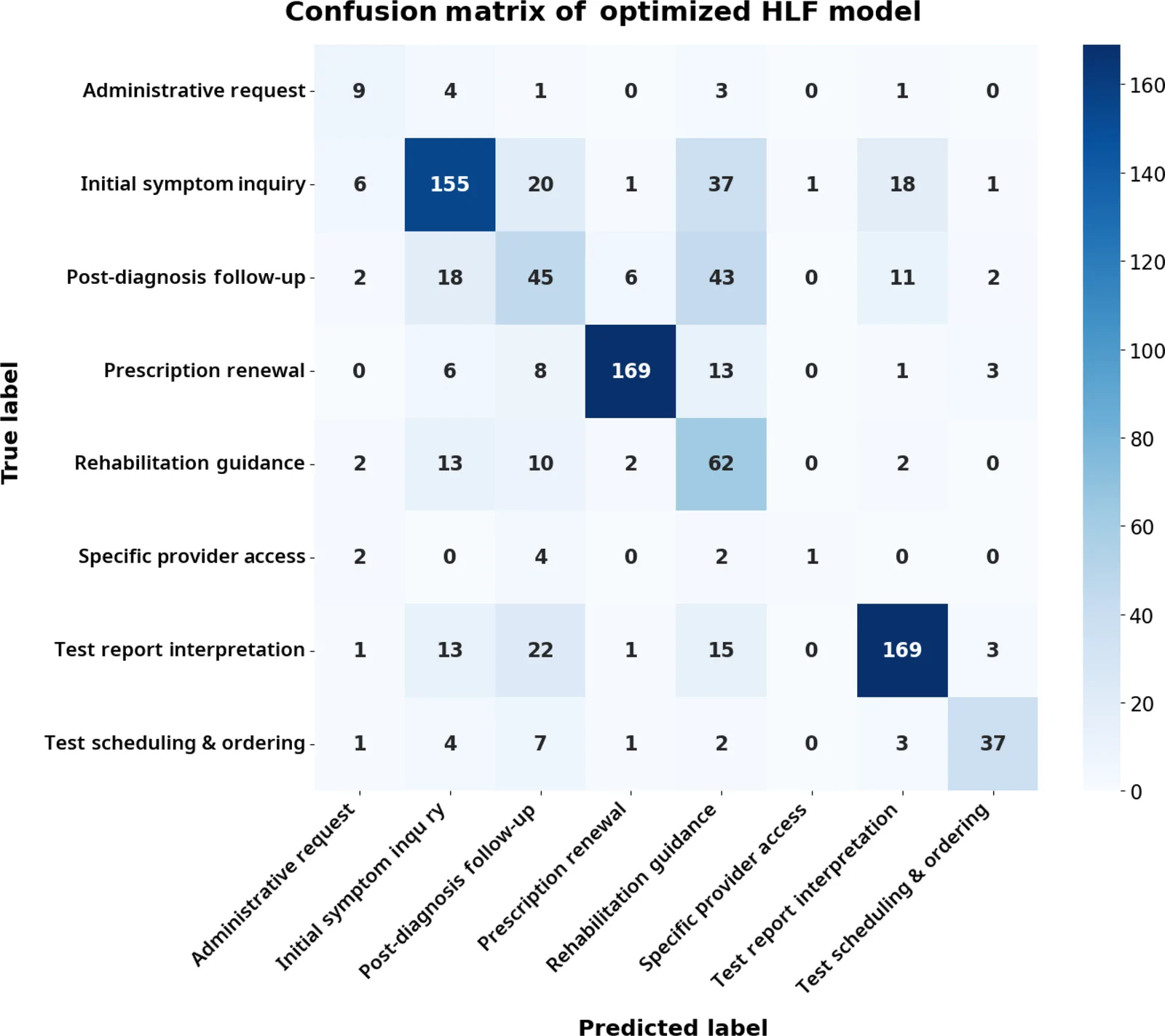
Confusion matrix of the hybrid learning framework (HLF) on the test set. The heatmap intensity reflects the number of samples in each cell. Diagonal elements represent correct predictions. Note the high accuracy for Prescription Renewal and the semantic confusion between Post-diagnosis Follow-Up and Rehabilitation Guidance.

High-performance categories: The model exhibited exceptional robustness in identifying structured and transactional intents. Specifically, for Prescription Renewal, the model achieved a precision of 0.94, correctly identifying 169 out of 180 test cases with minimal false positives. This high degree of reliability is important for potential automation, as errors in medication-related requests carry substantial clinical risk. Similarly, Test Report Interpretation showed strong performance with an F1-score of 0.79, successfully distinguishing requests to read lab results from general symptom inquiries.

Challenging categories: Performance dropped in categories characterized by high semantic ambiguity. The lowest performance was observed in Specific Provider Access, which yielded an F1-score of 0.18. This poor performance is largely attributed to the extreme scarcity of training samples (n=9 in the test set) rather than model deficiency. More critically, the confusion matrix revealed a persistent misclassification pattern between Post-diagnosis Follow-up (F1 = 0.37) and Rehabilitation Guidance (F1 = 0.46). Specifically, 32% of true Rehabilitation inquiries were misclassified as Follow-up. Qualitative analysis suggests that this error is not random. Postoperative follow-up visits frequently involve rehabilitation instructions, creating semantic overlap in patient descriptions. For example, the query “How should I move my leg after surgery?” could fit either category.

### Large-scale clinical deployment insights

Applying the validated HLF model to the full unlabeled dataset (N=28,851) allowed us to map the “Intent Fingerprints” of different clinical departments. Figure 5 illustrates the proportional distribution of patient needs across the top 10 specialties. The analysis revealed 3 distinct patterns of digital health utilization.

**Fig. 5.**
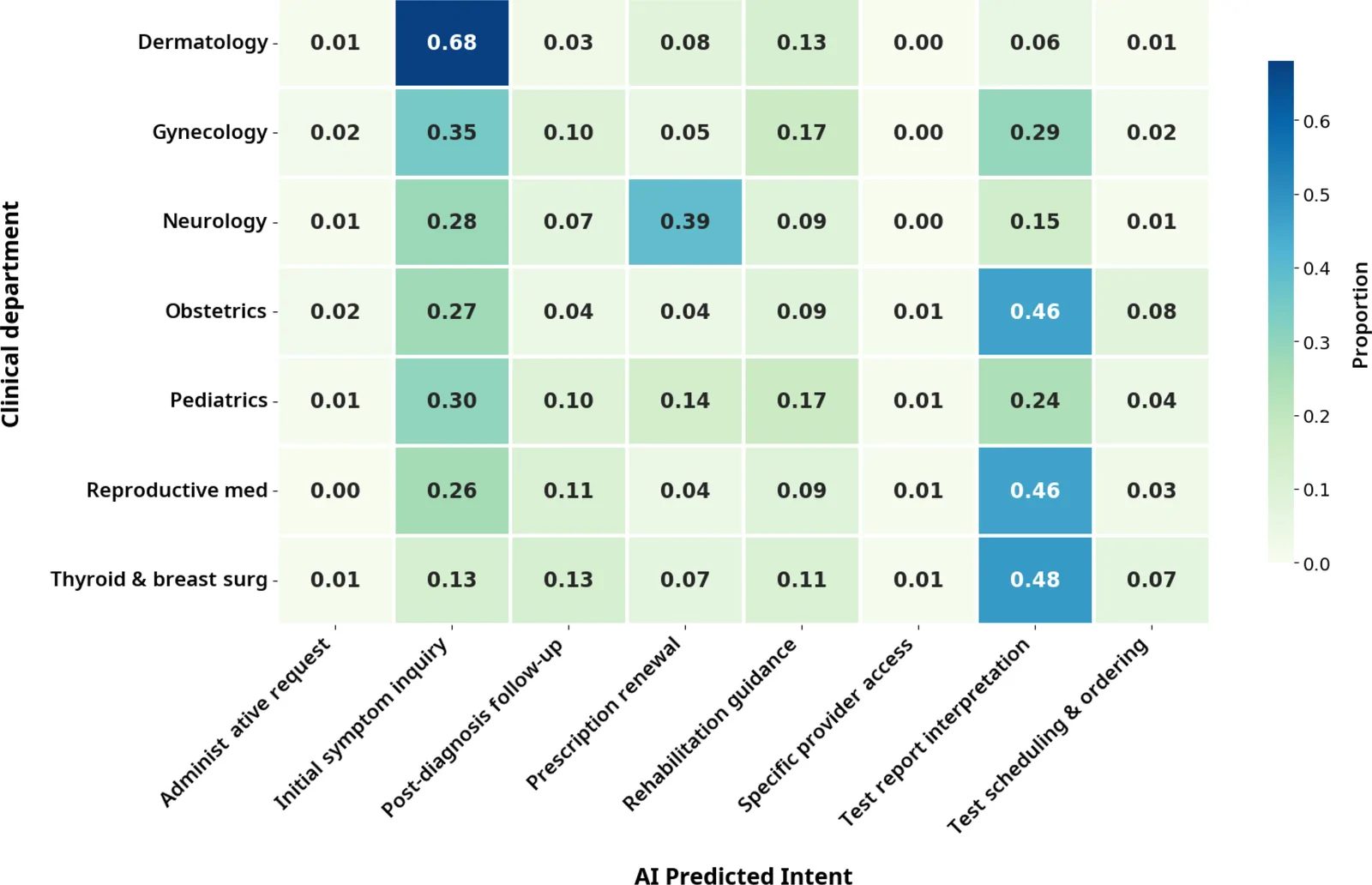
Heatmap of patient intent distribution across the top 10 clinical departments. Values indicate the row-normalized proportion of each intent within a department. Distinct patterns, such as the dominance of Initial Symptom Inquiry in Dermatology (0.68) versus Prescription Renewal in Neurology (0.39), highlight specialty-specific digital health needs.

First, the Chronic Maintenance Pattern was observed in departments such as Neurology and Cardiology. These specialties exhibited a high volume of Prescription Renewal requests, accounting for 39% and 35% of their total volume, respectively. This aligns with the chronic nature of conditions treated in these departments (e.g., epilepsy and hypertension), where long-term medication adherence is the primary driver of patient–physician interaction.

Second, the Diagnostic-Intensive Pattern was evident in Obstetrics and Reproductive Medicine. These departments showed a heavy concentration of Test Report Interpretation (>45%). This reflects the workflow of prenatal care and *in vitro* fertilization (IVF) cycles, which involve frequent monitoring of biomarkers (e.g., HCG and progesterone), generating a high volume of result-explanation requests from anxious patients.

Third, the Visual and Acute Care Pattern was seen in Dermatology and Pediatrics. These departments showed the highest proportions of Initial Symptom Inquiries (68% and 60%, respectively). In Dermatology, patients often upload photos for quick diagnosis, while in Pediatrics, parents frequently seek immediate advice for acute symptoms like fever or rash. These department-specific signatures validate the model’s external validity and offer a data-driven roadmap for hospital management to tailor digital services to the unique needs of each specialty.

## Discussion

### Principal findings and the “data-driven” paradigm

This study set out to address the dual challenge of taxonomy ambiguity and triage inefficiency in the rapidly expanding internet hospital ecosystem. Our primary contribution is the proof-of-concept validation of a closed-loop, data-driven framework that integrates unsupervised discovery with supervised detection, supported by external replication on an independent nationwide platform (Haodf, *N* = 497). Previous studies often relied on ad hoc administrative categories that suffer from definitional inaccuracies [29] and semantic discordance with lay patient terminology [30]. By contrast, our unsupervised clustering phase revealed a distinct semantic gap between these rigid taxonomies and how patients actually conceptualize their needs. For instance, we identified that patients conceptualize “Rehabilitation” and “Follow-up” as overlapping continua rather than discrete administrative events.

By operationalizing these findings into an HLF, we achieved a weighted F1-score of 0.680 on the primary dataset and 0.578 on an independent external platform, providing an initial performance reference that awaits further multicenter replication. The substantial performance gap (+28.7%) between our HLF and traditional SVMs confirms that medical intent classification requires deep semantic understanding beyond simple keyword matching. More critically, our work provides one of the first empirical comparisons between fine-tuned small language models and emerging LLMs in a real-world triage setting. This offers a pragmatic roadmap for hospitals navigating the “AI hype” cycle. This approach aligns with emerging methodologies that leverage unsupervised learning to uncover latent clinical phenotypes [31], demonstrating that hybrid frameworks can effectively bridge the data annotation gap [32].

### The “Specialist *vs.* Generalist” dilemma in medical AI

A notable finding is that, under zero-shot conditions, both the general-purpose Qwen2.5-7B and the domain-specific HuatuoGPT-II underperformed relative to the fine-tuned MacBERT model. This outcome is not surprising given the inherent asymmetry of the comparison—MacBERT was explicitly trained on the hospital’s data while the LLMs were not—but it highlights the practical value of task-specific adaptation for operational triage. This observation corroborates recent benchmarks showing that parameter scale does not guarantee domain-specific accuracy [33].

The hallucination of competence: While HuatuoGPT (F1 = 0.595) possesses vast encyclopedic knowledge (e.g., it knows the pharmacokinetics of Metformin), triage is not a knowledge retrieval task but a classification adherence task. LLMs often overthink or hallucinate categories, a phenomenon well-documented in recent safety evaluations of generative AI [34]. Furthermore, reliance on prompt engineering or zero-shot inference alone has shown inconsistent results in high-stakes clinical settings, whereas fine-tuning with domain-specific data has been demonstrated to be essential for achieving high accuracy in specialized medical tasks [35].

The efficiency of constraints: Our MacBERT model (0.1B parameters), though possessing less world knowledge, was constrained to learn the specific administrative logic of the hospital (e.g., only requests with refill keywords map to Prescription Renewal). This suggests that for specific, high-risk operational workflows, fine-tuned SLMs remain the gold standard for reliability, cost efficiency [36], and privacy compliance [37]. This finding challenges the prevailing narrative that bigger is always better in medical AI deployment.

### Clinical implications: From algorithms to operations

The ultimate value of an AI model lies in its ability to transform healthcare delivery. It is important to note that the operational strategies discussed below are inferred from the retrospective analysis of intent patterns and have not been evaluated in a prospective clinical setting. The predicted efficiency gains are therefore best viewed as hypotheses for future implementation studies rather than as demonstrated outcomes.

The “Intent Fingerprints” derived from our full-scale deployment (Fig. 5) offer tangible strategies for hospital administrators to move from reactive management to predictive resource allocation.

For chronic care (Neurology/Cardiology): The dominance of Prescription Renewal (39%) indicates that physicians in these departments are functioning largely as human rubber stamps for refills. Implementing our HLF as a prescreening agent could automatically route these requests to a fast-track queue, a strategy that, when carefully implemented, has been shown not to disrupt clinical workflow while improving prescription safety [38].

For diagnostic services (Obstetrics): The high volume of Test Report Interpretation (>45%) suggests a massive unmet need for information. This justifies the investment in Smart Lab Reports. Research indicates that direct, uninterpreted access to lab results is associated with significantly lower patient comprehension rates compared to traditional delivery methods [39] This creates a demand for AI-driven summarization—a task where adapted LLMs have been shown to generate summaries comparable or superior to those of medical experts for certain clinical texts [40].

For visual specialties (Dermatology): The 68% prevalence of symptom inquiries supports the prioritization of multimodal triage tools (Text + Image) over text-only chatbots for these departments. Text-only chatbots are insufficient. This aligns with findings in store-and-forward teledermatology, where diagnostic accuracy relies on visual data and can reach 80%, significantly outperforming text-only assessments by general practitioners [41]. These results advocate for multimodal fusion architectures, which have been shown to improve diagnostic accuracy over single-modality approaches [42]. These operational recommendations should be interpreted with the understanding that the HLF has not yet been deployed in a live clinical workflow. Quantifying the actual impact on waiting times, patient satisfaction, and clinician workload requires a prospective trial.

### Error analysis as a window into patient cognition

Our error analysis revealed that misclassifications were not random algorithmic failures but reflections of clinical ambiguity. The persistent confusion between Post-diagnosis Follow-up and Rehabilitation Guidance (32% error rate) highlights a misalignment between the hospital’s administrative structure and the patient’s journey. This underscores the need for Service Design thinking in healthcare operations. Patients often ask, “Can I walk now?” Is this a rehabilitation question or a post-op recovery check?

This insight suggests that improving AI performance further may not require better algorithms, but better service design. Hospitals should consider merging these categories into a unified Post-Acute Care track, simplifying the patient interface while maintaining backend granularity. This underscores the potential of AI not just to automate existing processes, but to audit and redesign the care processes themselves.

In conclusion, this study presents a rigorous, end-to-end framework for demystifying patient needs in internet hospitals, with initial evidence of cross-platform generalizability from an independent external validation dataset (*N* = 497). By moving beyond arbitrary administrative labels to a data-driven taxonomy, we revealed the nuanced reality of online medical seeking behavior. Technically, we demonstrated that zero-shot inference from LLMs, even those pretrained on biomedical corpora, is insufficient for reliable hospital triage in the studied setting. Task-specific fine-tuning—here applied to a compact MacBERT model—substantially improved classification accuracy compared to zero-shot LLM baselines. However, these findings reflect the value of task adaptation rather than an inherent superiority of small architectures over large ones; whether fine-tuned LLMs would achieve further gains remains to be investigated.

Clinically, our large-scale deployment unmasked the distinct operational fingerprints of different specialties within the studied institution, illustrating how data-driven demand profiling could inform digital service design. The specific intent distributions observed should be regarded as a methodological template rather than directly transferable benchmarks. As healthcare continues its digital transformation, the HLF approach demonstrates that hybrid unsupervised-to-supervised pipelines can decode patient intent without reliance on preexisting administrative taxonomies. It further shows that fine-tuned task-specific models transfer more robustly across platforms than zero-shot LLMs. However, the generalizability of this single-center, Chinese-language evidence remains to be fully established. Multicenter, cross-cultural prospective studies are needed to determine whether the framework’s advantages hold across diverse healthcare systems and digital platforms. Pending such evidence, these findings should be interpreted as a methodological advance with promising external validation rather than as a universally deployable solution.

Several limitations warrant consideration. First, the dataset was sourced exclusively from a single tertiary center in China and is limited to Chinese-language patient inquiries. While this ensures internal consistency of the annotation schema, the specific intent distribution may differ in community hospitals, across distinct cultural settings, or in non-Chinese-language digital health platforms, potentially limiting the external validity of our taxonomy [43]. Future work should aim to validate these findings across multicenter cohorts, potentially utilizing privacy-preserving and fairness-aware federated learning frameworks to aggregate insights without compromising institutional data privacy [44].

Second, our current HLF model relies solely on textual input, which may be insufficient for visual-centric specialties. As indicated by the high volume of symptom inquiries in Dermatology, visual cues are often diagnostic prerequisites. Integrating image encoders (e.g., vision transformers) into a multimodal architecture represents a critical frontier for enhancing triage accuracy in these specific domains.

Third, while our retrospective analysis provides strong evidence for operational efficiency, it does not measure real-time clinical outcomes. The calculated time-savings are theoretical simulations based on historical logs. A prospective randomized controlled trial is the necessary next step to quantify the actual impact on physician workload, patient waiting times, and satisfaction scores in a live clinical environment [45].

Fourth, the evaluation relied on a single stratified train–test split. While bootstrap confidence intervals were reported to quantify the stability of the point estimates, this approach does not fully capture model variance across alternative data partitions. Future work should incorporate *k*-fold cross-validation to provide more robust performance bounds.

## Method

### Study design and data source

This study employed a retrospective, exploratory sequential mixed-methods design. The initial qualitative phase used expert review of cluster-derived TF-IDF keywords to develop the 8-category intent taxonomy; this taxonomy then served as the annotation schema for constructing the gold-standard labeled dataset. The subsequent quantitative phase trained and evaluated the supervised MacBERT classifier on these labels. This sequential structure ensured that the classification schema was grounded in the natural semantic organization of patient inquiries before any quantitative modeling was performed. The data source was the online inquiry platform of a large tertiary public hospital in Guangdong, China. This platform serves as a digital front door for patients, allowing them to submit textual descriptions of their symptoms or administrative needs to specific departments. We extracted all historical interaction records spanning a 12-month period from 2023 January 1 to 2023 December 31. The initial raw corpus contained 28,851 records.

### Data preprocessing and privacy protection

To ensure the high quality of the dataset required for training deep learning models, a multistage preprocessing pipeline was implemented. First, we removed duplicate entries caused by system glitches or repeated submissions by the same user within a short timeframe. Second, we filtered out system-generated automated responses (e.g., “The doctor is currently busy”) to retain only patient-generated content. Third, inquiries containing fewer than 5 Chinese characters (e.g., “Hello” and “Are you there?”) were excluded, as these brief texts typically lack sufficient semantic context for meaningful intent classification.

Ethical considerations were prioritized throughout the study. Although the study was retrospective and observational, we strictly adhered to privacy protection protocols. All personally identifiable information—including patient names, physician names, phone numbers, and identification numbers—was rigorously desensitized using regular expression matching and named entity recognition techniques prior to any analytical processing. The study protocol was reviewed and approved by the Ethics Committee of Yuebei People Hospital (Approval No. YBSKY-2025-123-001), with a waiver of informed consent due to the noninterventional nature of the study.

### External validation dataset

To empirically assess cross-institutional generalizability, we constructed an independent external validation dataset from Haodf (www.haodf.com), a nationwide public online medical consultation platform distinct from the WeChat-based hospital portal used in the primary study. Haodf serves patients across multiple hospitals and geographic regions through a web-based interface, providing a meaningful test of domain shift in platform type, patient population, and inquiry style. A total of 497 patient-initiated inquiries were collected and independently annotated using the identical 8-category taxonomy developed on the primary dataset. Inter-annotator agreement, assessed by Cohen’s Kappa, was 0.88, confirming that the taxonomy transferred across platforms without requiring redefinition. The intent distribution in the external dataset differed from that of the primary dataset, reflecting the distinct user populations and use cases of the 2 platforms. These distributional differences provide a meaningful test of the model’s robustness to domain shift.

### Taxonomy development and gold-standard construction

Conceptual framework: Defining “Patient Intent”. In this study, “patient intent” refers to the actionable purpose motivating a patient’s online inquiry—specifically, the clinical or administrative need that would determine appropriate departmental routing in a real triage scenario. This operational definition deliberately encompasses clinical (e.g., symptom reporting), administrative (e.g., appointment scheduling), and transactional (e.g., Prescription Renewal) elements, reflecting the reality that patients navigating internet hospitals do not distinguish among these categories when articulating their needs.

Conceptually, this multidimensional framing is consistent with prior taxonomies developed for patient-generated messages in digital health settings. Cronin *et al.* [46] classified patient portal messages into informational, medical, logistical, and social categories, with subcategories including prescriptions, appointments, problems, tests, and follow-up—a structure that mirrors several core categories of our 8-category taxonomy. Notably, these authors observed that portal messages routinely contain more than one communication type, reinforcing our finding that patient inquiries span clinical and administrative boundaries. In a related effort, Ren *et al.* [47] demonstrated that the informal, colloquial language of patient-generated messages benefits substantially from models pretrained on corpora with matching linguistic style—a finding that underscores the gap that standardized clinical terminologies such as the International Classification of Diseases (ICD-10) and the Nomenclature of Medicine Clinical Terms (SNOMED CT) were not designed to fill. The 8-category taxonomy developed in this study thus serves a complementary function: it maps the digital front door rather than the clinical encounter itself.

Unlike previous studies that relied on predefined administrative categories, we adopted a data-driven approach to discover the natural structure of patient needs.

Unsupervised discovery: We utilized the BERT (bert-base-Chinese) model to generate semantic embeddings for the entire unlabeled corpus. BERT’s bidirectional attention mechanism allows it to capture the nuanced context of medical queries better than traditional bag-of-words models. We then applied K-Means clustering to these embeddings. The optimal number of clusters (k=8) was rigorously determined using the Elbow Method, identifying the point where the marginal gain in variance explanation diminished. This structure was further validated visually using t-SNE.

Annotation guidelines and process: Based on the cluster-level TF-IDF keywords and representative exemplars extracted from each cluster, 2 domain experts (a senior attending physician with 10+ years of clinical experience and a medical informatics researcher) jointly drafted a preliminary coding scheme comprising 8 mutually exclusive intent categories with written definitions and inclusion/exclusion criteria for each category. This scheme was iteratively refined through independent pilot annotation of 500 randomly sampled records. For instance, a clear operational distinction was drawn between Post-diagnosis Follow-up (routine checks scheduled after a known procedure or diagnosis, e.g., “I had a thyroidectomy last month, should I come back for a checkup?”) and Rehabilitation Guidance (specific inquiries about functional recovery exercises or lifestyle modifications, e.g., “What exercises should I do after my knee surgery?”).

Critically, the final annotation was performed without access to the cluster assignments of individual records. Although cluster-level keywords informed the initial drafting of category definitions, annotators were blinded to which cluster any specific inquiry originated from during labeling. This design ensures that the gold-standard labels reflect human clinical judgment rather than recapitulating the unsupervised clustering output, thereby mitigating the risk of circularity between taxonomy discovery and supervised model training.

For inquiries expressing multiple intents simultaneously (e.g., a patient describing a new symptom while also requesting a prescription refill), annotators were instructed to identify the primary actionable intent—defined as the request that would determine the appropriate departmental routing in a real triage scenario. If no single intent was clearly dominant (adjudicated by a hierarchical decision rule prioritizing clinical urgency over administrative need), the inquiry was flagged for consensus discussion rather than being assigned a default label. In total, multi-intent queries accounted for approximately 9% of the annotated sample; all were resolved through the consensus procedure described below.

Inter-annotator agreement: A subset of 4,814 inquiries was randomly sampled from the full corpus for the final gold-standard dataset. Two trained annotators independently labeled all records using only the written category definitions; neither annotator had access to the cluster assignments or to each other’s labels during the initial annotation phase. To assess annotation reliability, we calculated Cohen’s Kappa coefficient. The initial agreement was 0.88, indicating strong consistency.

Discrepant labels were resolved through a structured 3-stage protocol rather than informal discussion. First, each annotator independently re-reviewed the disputed record against the written category definitions. Second, if disagreement persisted, both annotators jointly examined the record alongside a third senior clinical expert (the arbitrator), who facilitated a structured discussion with reference to the operational definitions and the hierarchical decision rule for multi-intent queries described above. Third, the final label was assigned by majority agreement; if no majority emerged, the record was excluded from the gold-standard dataset. The final number of records excluded via this process was fewer than 10 records (<0.2%). The entire arbitration process was documented with a time-stamped log to maintain an auditable annotation trail.

A potential concern in any 2-stage framework that derives a taxonomy from unsupervised clustering and subsequently trains a supervised classifier on the same data source is circularity: the supervised model may learn to reproduce the clustering output rather than capture clinically meaningful distinctions. We addressed this concern through several design choices. First, the taxonomy was defined through expert refinement of cluster-derived keywords, not by directly adopting cluster assignments as labels; the 8 categories represent clinically motivated abstractions rather than algorithmic outputs. Second, annotators were blinded to individual cluster assignments during labeling, ensuring that the gold-standard labels encode human clinical judgment rather than cluster membership. Third, the supervised classifier was trained on the annotator-assigned labels, not on cluster IDs, and its performance was measured against a held-out test set of human annotations—not against agreement with the original clusters. Had the supervised model merely recapitulated the clustering, its performance ceiling would have been bounded by the fidelity of the clustering itself; the observed inter-annotator agreement (κ = 0.88) and the model’s ability to exceed traditional baselines by substantial margins suggest that the learned representations capture signal beyond the initial unsupervised partition.

### Model development: The HLF

We proposed an HLF that leverages the strengths of pretrained language models.

Model architecture: We utilized MacBERT-base [21] as the backbone encoder. MacBERT improves upon the original BERT by employing a whole word masking strategy and N-gram masking, which is particularly effective for Chinese medical texts where medical entities often span multiple characters. A fully connected classification layer was added on top of the [CLS] token representation.

Training configuration: The model was implemented using the PyTorch and HuggingFace Transformers libraries. We fine-tuned the model using the AdamW optimizer with a linear learning rate scheduler (initial learning rate = 3e−5). The batch size was set to 16, and the maximum sequence length was truncated or padded to 128 tokens. To mitigate overfitting, we employed an early stopping mechanism: training was halted if the validation loss did not improve for 3 consecutive epochs, with the maximum epochs set to 10.

Unclassifiable inquiries: Of the 28,851 records in the full preprocessed corpus, a subset could not be reliably assigned to any of the 8 intent categories during the annotation pilot. These comprised primarily (a) purely social exchanges (e.g., “Thank you, doctor” and “Goodbye”), (b) incomplete or truncated messages, and (iii) platform navigation queries unrelated to clinical care (e.g., “How do I upload an image?”). Because these records contained no actionable clinical or administrative intent, they were excluded from the gold-standard dataset. An explicit “Others” category was not adopted, as its inherent heterogeneity would have introduced a catch-all class with no operational relevance to departmental routing—the primary clinical use case of the HLF. The 4,814 annotated records therefore represent the classifiable subset of the corpus.

### Baselines and comparative analysis

To position our HLF within the broader landscape of NLP technologies, we compared it against 3 distinct tiers of baselines:

1. Traditional machine learning: As a performance baseline, we implemented SVM classifiers. Text features were extracted using TF-IDF, keeping the top 4,814 most frequent vocabulary terms.

2. General LLMs: We selected Qwen2.5-7B-Instruct to represent state-of-the-art open-source LLMs. The model was evaluated in a zero-shot setting to test its generalization capability.

3. Medical-specific LLMs: We employed HuatuoGPT-II (7B), an LLM pretrained on massive Chinese biomedical corpora, to benchmark whether domain-specific pretraining alone is sufficient for hospital triage without fine-tuning.

Prompt engineering: For both LLMs, we designed a structured prompt to enforce deterministic classification: “Role: You are a professional medical triage assistant. Task: Classify the following patient inquiry into exactly one of the eight categories: [List]. Input: {text}. Output: Category Name Only”. Inference was conducted with a temperature setting of 0.01 to minimize hallucinations.

We note that the comparison between fine-tuned MacBERT and zero-shot LLMs is asymmetric by design: the former benefits from task-specific training on the target hospital’s data, while the latter are evaluated out-of-the-box. This design choice reflects a pragmatic clinical deployment question—whether a hospital with limited annotation resources should invest in fine-tuning a small model or rely on a general-purpose LLM without task adaptation. However, it does not capture the full capability of LLMs, which can be substantially improved through few-shot prompting or domain-specific fine-tuning. Our conclusions are therefore limited to the zero-shot setting and should not be interpreted as a general claim that small models inherently outperform LLMs.

### Statistical analysis and hardware

All experiments were conducted on a high-performance workstation equipped with an NVIDIA RTX 3090 GPU (24 GB VRAM) and 64 GB of system RAM.

The gold-standard dataset (*N* = 4,814) was partitioned into a training set (80%, *n* = 3,851) and a held-out test set (20%, *n* = 963) using stratified sampling to preserve the original class distribution. An internal validation subset (10% of the training set, *n* = 385) was used for early stopping; the test set was not used for model selection and was evaluated only at the conclusion of training. Given the inherent class imbalance in medical data (e.g., Prescription Renewal is much more frequent than Specific Provider Access), we reported the weighted F1-score as the primary metric for model comparison. To address class imbalance without distorting the natural consultation distribution—which itself carries clinically relevant signal about patient demand patterns—we relied on stratified sampling for the train–test split and adopted weighted averaging for all reported F1-scores, which proportionally accounts for class frequency. No resampling or synthetic augmentation was applied, as the observed long-tail distribution reflects genuine variation in patient help-seeking behavior. Confusion matrices were generated to visualize and analyze the specific error patterns between clinically similar categories. To assess metric stability, 95% confidence intervals for weighted F1-scores were estimated via bootstrap resampling (1,000 iterations). Pairwise model comparisons were assessed using McNemar’s test with continuity correction. *K*-fold cross-validation was not performed due to the computational cost of fine-tuning a transformer-based model across multiple data partitions (each fold requiring training from scratch); bootstrap resampling of test-set predictions provides an estimate of metric stability without requiring model retraining.

## Data Availability

The primary hospital dataset (*N* = 4,814) cannot be publicly released in full due to restrictions imposed by the institutional data sharing agreement. To facilitate reproducibility, we provide de-identified exemplar records (2 to 3 per intent category) and a statistical summary of per-category sample counts and text lengths in the accompanying GitHub repository (https://github.com/wenshilaohu/hlf-triage). The complete independent external validation dataset sourced from the Haodf public platform (*N* = 497) is provided as a list of source URLs with corresponding intent labels. The full primary dataset remains available upon request to the corresponding author, subject to institutional ethics committee approval and a data use agreement. The complete codebase for the HLF, including data preprocessing, BERT embedding generation, K-Means clustering, the MacBERT fine-tuning pipeline, and inference scripts for all baseline models, is publicly available at https://github.com/wenshilaohu/hlf-triage. The repository includes a README with environment configuration details, hyperparameter settings, and fixed random seeds (seed = 42) to ensure deterministic reproducibility. This study utilized the following open-source software and libraries, which are publicly available: text preprocessing and analysis were performed using Jieba for Chinese word segmentation and the Transformers library (Hugging Face) for BERT embeddings and model fine-tuning. Unsupervised clustering was conducted using scikit-learn, and statistical analyses were performed with SciPy and pandas. Visualization was created using Matplotlib and Seaborn.
